# Efficacy and mechanisms of a single-session behavioral medicine class among patients with chronic pain taking prescription opioids: study protocol for a randomized controlled trial

**DOI:** 10.1186/s13063-020-04415-x

**Published:** 2020-06-12

**Authors:** Maisa S. Ziadni, Abby L. Chen, Tyler Winslow, Sean C. Mackey, Beth D. Darnall

**Affiliations:** 1 Department of Anesthesiology, Perioperative and Pain Medicine, Stanford University School of Medicine, Stanford University, 1070 Arastradero Road, Suite 200, Palo Alto, CA 94304 USA; 2 Division of Pain Medicine, Stanford Systems Neuroscience and Pain Laboratory, Stanford University School of Medicine, 1070 Arastradero Road, Suite 200, MC 2C2728, Palo Alto, CA 94304 USA

**Keywords:** Chronic pain, Prescription opioids, Pain catastrophizing, Cognitive-behavioral therapy, Behavioral medicine, Treatment

## Abstract

**Background:**

Independent of pain intensity, pain-specific distress is highly predictive of pain treatment needs, including the need for prescription opioids. Given the inherently distressing nature of chronic pain, there is a need to equip individuals with pain education and self-regulatory skills that are shown to improve adaptation and improve their response to medical treatments. Brief, targeted behavioral medicine interventions may efficiently address the key individual factors, improve self-regulation in the context of pain, and reduce the need for opioid therapy. This highlights the critical need for targeted, cost-effective interventions that efficiently address the key psychological factors that can amplify the need for opioids and increased risk for misuse. In this trial, the primary goal is to test the comparative efficacy of a single-session skills-based pain management class to a health education active control group among patients with chronic pain who are taking opioids.

**Methods/design:**

Our study is a randomized, double-blind clinical trial testing the superiority of our 2-h, single-session skills-based pain management class against a 2-h health education class. We will enroll 136 adult patients with mixed-etiology chronic pain who are taking opioid prescription medication and randomize 1:1 to one of the two treatment arms. We hypothesize superiority for the skills-based pain class for pain control, self-regulation of pain-specific distress, and reduced opioid use measured by daily morphine equivalent. Team researchers masked to treatment assignment will assess outcomes up to 12 months post treatment.

**Discussion:**

This study aims to test the utility of a single-session, 2-h skills-based pain management class to improve self-regulation of pain and reduce opioid use. Findings from our project have the potential to shift current research and clinical paradigms by testing a brief and scalable intervention that could reduce the need for opioids and prevent misuse effectively, efficiently, and economically. Further, elucidation of the mechanisms of opioid use can facilitate refinement of more targeted future treatments.

**Trial registration:**

ClinicalTrials.gov, ID: NCT03950791. Registered on 10 May 2019.

## Background

There is a critical need for reduced emphasis on high-risk pain treatments and better integration of behavioral medicine and self-management strategies to treat pain comprehensively by integrating a “whole person” approach to pain care [1–3]. To date, the US lacks scalable behavioral medicine for pain thereby underscoring the need for solutions that are accessible, low-cost, and low-burden. Evidence-based, skills-based behavioral medicine for pain has been shown to reduce pain-specific distress [4, 5], pain intensity [6], pain bothersomeness [7], improve response to pain treatments [8], and reduce opioid use among perioperative patients [9].

Inadequate treatment of chronic pain is an interrelated public health crisis [10, 11]. An Institute of Medicine Pain Report noted that chronic pain affects ~ 100 million American adults and costs US$635 billion annually [1]. Opioid prescribing continues to fuel the epidemic as one of the most commonly used treatments for chronic pain [12]. The prevalence of prescription opioid use increased from 4.1% of US adults in 1999–2000 to 6.8% in 2013–2014 [13], leading to sharp increases in opioid abuse and accidental overdose [12, 14, 15]. Consequently, both public health crises are pervasive and costly in economic and human terms. Because chronic pain is often treated with opioids, the two crises intersect with bidirectional relationships. Solutions to one frequently and directly influence the other.

To date, little research has examined the efficacy of skills-based interventions in patients taking long-term opioid therapy. Cognitive-behavior therapy (CBT) has emerged with preliminary promising results for opioid-treated chronic pain, with reductions in opioid use [16], misuse [17], and aberrant opioid-related behaviors [18]. Improved self-regulation of pain and pain-specific distress (i.e., pain catastrophizing, depression, anxiety) is most commonly achieved with eight sessions of group cognitive behavioral therapy (pain CBT; 16 h of treatment time) [19]. While longer-course multi-session pain CBT modalities are effective, patients incur many barriers including substantial cost, time, and travel burden, lack of local skilled clinicians, insurance coverage, and co-payment costs [20–22]. These barriers impair broad access to skills-based behavioral medicine for chronic pain, promote a biomedical treatment approach, and may promote pharmacological and interventional modalities as the only options available to patients.

A single-session, 2-h skills-based pain management class (“Empowered Relief” (ER)) was shown to reduce pain-specific distress and improve self-regulation at 4-week follow-up in a cohort of 57 mixed-etiology chronic pain patients receiving treatment at a tertiary referral, multidisciplinary chronic pain clinic [4]. A recent randomized controlled trial showed that a digital version of the class, adapted to the perioperative setting, effectively enhanced time to opioid cessation after breast cancer surgery compared to a digital health education control (“My Surgical Success”) [9]. Importantly, neither “Empowered Relief” nor “My Surgical Success” direct patients to use less opioid medication. For the first time, the current study seeks to test the impact of “Empowered Relief” on opioid use in adults with mixed-etiology chronic pain who are taking long-term opioid therapy.

### Specific aims

Our two specific aims and their corresponding hypotheses are outlined below:

1. We will conduct a randomized controlled trial comparing the single-session skills-based pain management class to a single-session health education control (HE) (no actionable skills)
  Hypothesis 1a: the single-session skills-based pain management class will lead to greater reductions in opioid use compared to the HE class
  Hypothesis 1b: the single-session skills-based pain management class will lead to greater reductions in opioid misuse, pain-related distress (pain catastrophizing, depression, anxiety), and pain interference compared to the HE class
2. To characterize the influence of daily pain-catastrophizing on same-day and next-day opioid use
  Hypothesis 2a: daily pain-catastrophizing will predict same-day and next-day opioid use. Relationships between daily pain-catastrophizing and same-day and next-day opioid use are reduced in the ER class compared to HE
  Exploratory Hypothesis 2b: daily mean changes in pain catastrophizing (baseline to 3 months post treatment) will predict reduction in opioid use, opioid misuse, and mean change in pain and function measures in the single-session skills-based pain management class at 3, 6, and 12 months post treatment

## Methods/design

### Overview

**Fig. 1:**
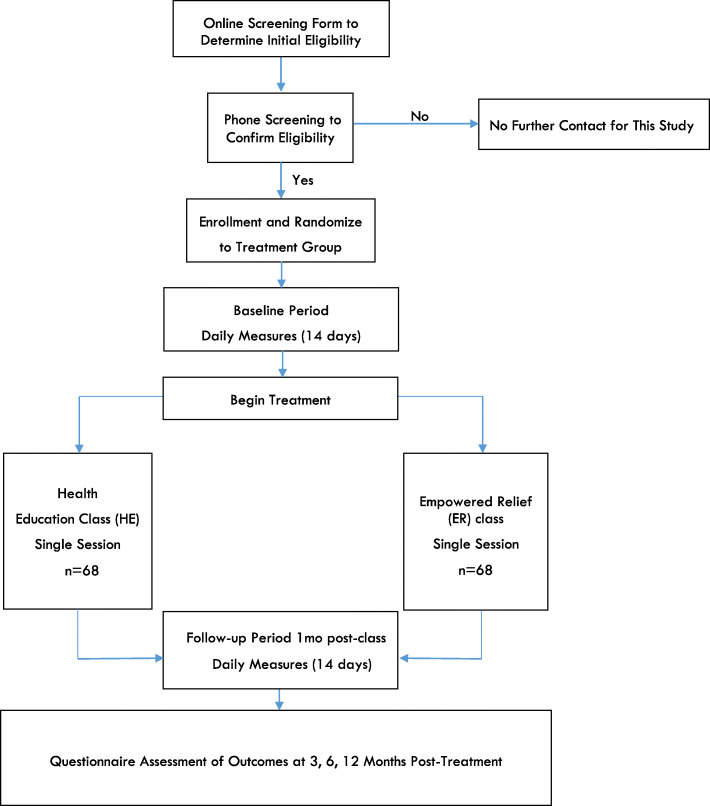
Flowchart of the trial protocol

**Fig. 2:**
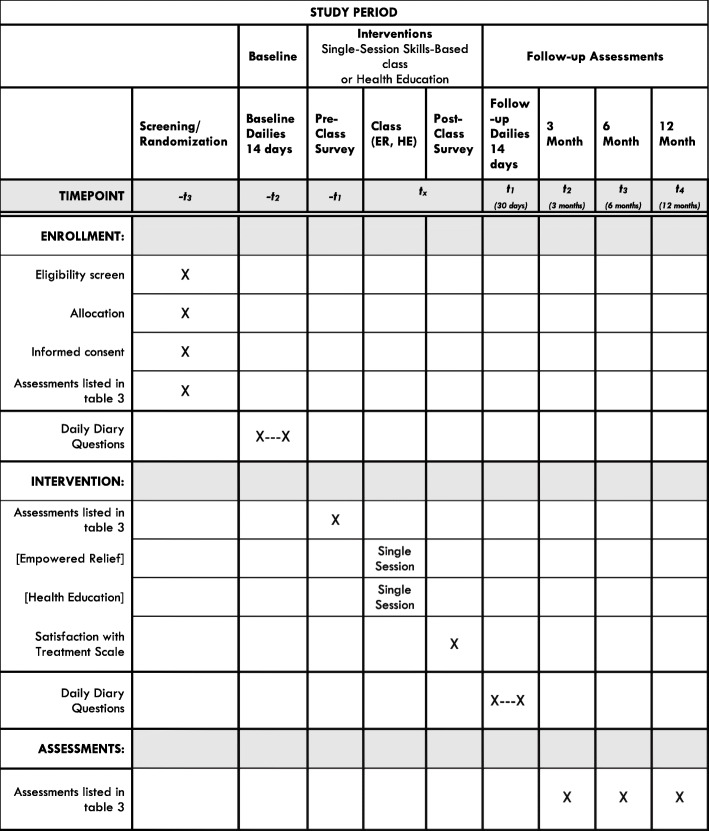
The schedule of enrollment, interventions, and assessments

We are conducting a randomized clinical trial in which individuals with a chronic pain condition who are currently taking prescription opioids are randomly assigned to one of two arms: a single-session skills-based pain management class or a single-session HE class (active control; control group) (Figs. 1 and 2). Participants will be followed for 12 months after treatment. Participants will be assessed via an online screening form, a telephone screening, enrollment survey, pre-class survey, a 2-week daily baseline period, 2-week daily follow-up period, and at 3, 6, and 12 months post treatment. Team statisticians blinded to participant treatment assignment will assess outcomes 3, 6, and 12 months after treatment. The primary outcome is opioid use 3 months post treatment. Secondary outcomes include reductions in pain-specific distress, pain interference and opioid misuse at 3 months.

The protocol for this trial has been approved by the Stanford Institutional Review Board (IRB). All participants will be required to give informed consent to a trained study team member prior to enrollment in the study.

### Study sample and setting

Participants for this trial will be recruited through targeted emails and advertisements at Stanford’s pain management clinics, in addition to the Stanford Systems Neuroscience and Pain Laboratory (SNAPL) database. Recruitment efforts will extend to social-media marketing, and local advertisements in clinics and in the community. All advertisements will direct interested individuals to an online screening form that assesses for initial eligibility. The study will enroll 136 adults (age 18–80 years) with a chronic, non-cancer pain condition, currently using prescription opioids of a ≥ 20 morphine-equivalent daily dose (MEDD) for ≥ 3 months and who meet the study criteria (Table 1). The sample size accounts for expected attrition. Eligibility will be assessed by the research staff.

**Table 1:** Inclusion criteria

Inclusion criteria	Rationale	Sources
Pain > 6 months more than half the time	Study restricted to non-cancer chronic pain	A,TS
Currently using prescription opioids with a morphine-equivalent daily dose (MEDD) of ≥ 20 mg	Significant level of opioid use to treat and detect meaningful reduction	A,TS
Opioid use duration ≥ 3 months	Definition of chronic opioid use	A,TS
English fluency	Ability to complete study procedures	A,TS
Men and women 18 to 80 years of age		A,TS

*A* automated data gathered from REDCap surveys, *TS* telephone screening

### Inclusion and exclusion criteria

Tables 1 and 2 list the inclusion and exclusion criteria, respectively, as well as the rationale for each criterion and the sources where each criterion will be assessed. Patients who are taking non-opioid analgesic medication (e.g., gabapentin, lyrica) will not be excluded from the study. Additionally, we require that the participants be willing and available to participate in the full treatment session to which they are assigned and able to respond to the daily measures (at baseline and follow-up) and post treatment (3, 6, and 12 months) follow-up questionnaires (Table 3).

**Table 2:** Exclusion criteria

Exclusion criteria	Rationale	Sources
Open litigation regarding a medical condition	Source of bias	A,TS
Inability to provide informed consent and complete study procedures	Not able to complete study procedures	A,TS, E
Active participation in CBT-based treatment	Possible bias due to current exposure to treatment groups	A,TS
Active suicidality		E

*A* automated data gathered from REDCap surveys, *TS* telephone screening, *E* enrollment survey

**Table 3:**
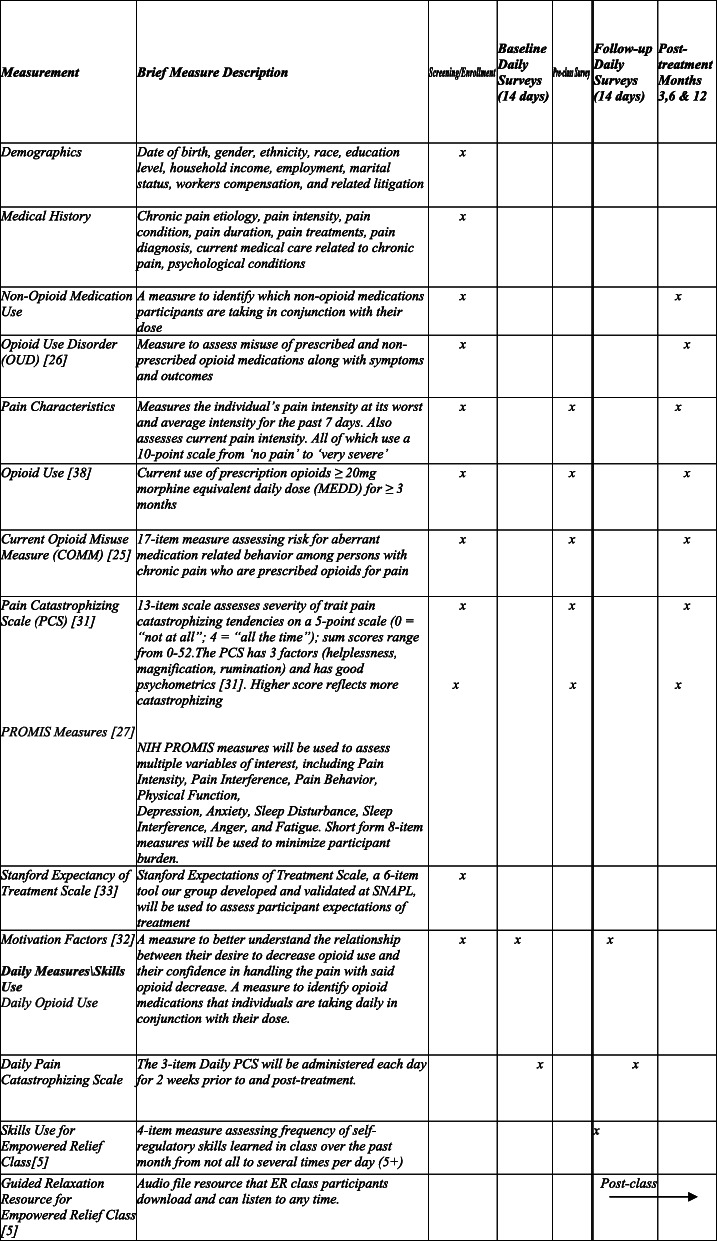
Baseline and follow-up measures

### Recruitment procedures

Recruitment will occur in waves with recruitment windows open for the 6 weeks preceding each scheduled class. Pre-treatment, 2-week daily surveys will be administered following enrollment and must be initiated at least 2 weeks prior to the scheduled class to allow for completion. Participants must attend their assigned class within 4 weeks of completing the pre-treatment daily surveys.

Because the study intervention involves group treatment classes, we are recruiting participants in cohorts consisting of 7–12 participants per class cohort (minimum of 4 participants, maximum of 15 participants per cohort) for both treatment arms.

Interested individuals deemed initially eligible by the online screening will be further screened over the telephone. Eligible individuals will then be invited to enroll in the study, and consented with a research staff over the telephone, after which they provide an electronic signature to the consent form emailed to them. Participants are randomized following eligibility confirmation and informed consent procedures. Then participants complete the enrollment survey, which includes information related to their chronic pain, opioid use, non-opioid medication use, medical history, and psychosocial wellbeing. Additionally, measures of opioid-misuse behaviors and severity, treatment expectancies and patient motivation factors will be administered.

### Randomization

Enrolled participants will be 1:1 randomized to one of two treatment arms: the ER and the chronic pain HE class. No blocking or stratification will be utilized. An automated program in REDCap will randomly assign a participant to a treatment arm when enrolled and will ensure blinded randomization, as well as equal numbers in both arms at the end of data collection.

### Blinding

Participants cannot, and will not, be blinded to the intervention that they are randomized to. A clinical psychologist who will be trained in the intervention, blind to participants and who has no involvement in data analysis will deliver the intervention. The study coordinator will be responsible for handling the randomization process through REDCap but will remain blinded to the randomization scheme. All data given to the statistician will be blinded, except as required when reporting adverse events (AEs). All research data will be kept separate from identifiers and linked using a participant number. An alternative research team member will have access to the data and will be responsible for the data monitoring. Only the principal investigators (PIs) will have access to the file linking names and participant numbers and the file will be stored in their locked offices. The team will have access to the final unidentified dataset.

### Study treatments

Both study arms (ER and HE) consist of a single-session, 2-h group class. Participants will leave the ER class with home-based resources that facilitate ongoing self-regulation and pain self-management.

#### Single-session skills-based pain management class (ER)

Our group developed the single-session skills-based pain management class in 2013 with a goal of rapidly equipping patients with skills to self-regulate pain-specific distress. Pilot data revealed significantly reduced pain-specific distress – as indexed by reductions in pain catastrophizing – 1 month post treatment regardless of comorbid depression and anxiety [4]. The single-session skills-based pain management class (ER) is also the subject of a National Institutes of Health (NIH)-funded randomized controlled trial in chronic low-back pain [5] with pain reduction as the primary outcome.

For this study, a clinical psychologist trained in delivering the 2-h intervention will administer the class to groups of enrolled participants. Brief education on opioid reduction is included in the class, along with a one-page handout that summarizes key research findings. Materials are already in use in our existing projects [23]. The class is delivered by PowerPoint presentation and includes mind-body pain science, the importance of self-regulation in the context of pain and stress, and evidence-based skills that target pain-specific distress and enhance pain control. Participants will be guided in developing their own self-treatment plan and acquiring the skills necessary to decrease pain- and stress-related physiological hyperarousal, and to enhance the regulation of cognition and emotion within the context of pain. At the end of the class, participants will leave with: (1) a self-tailored personalized plan to target pain-specific distress; (2) a 20-min guided-relaxation response electronic app; and (3) a printed copy of the didactic class content. The app “Body Mind Medicine 2.0” was developed by the Stanford University IT team and offered as a free app for participants in the intervention arm. It includes a 20-min guided binaural relaxation resource developed by a member of the research team. Study staff will guide participants through downloading the app on their smart phone, and they will be provided with a unique user ID. App use data are passively collected and will be tracked through REDCap. Participants are encouraged to use the app frequently, but no additional instruction is provided.

Cohort effects are expected to be minimal due to the single-session nature of the class, the class content is highly didactic in nature, and because participant interaction is relatively minimal. However, we will examine cohort effects and instructor effects as potential covariates.

#### Health education class

This is a 2-h class that will be delivered by a health educator using a PowerPoint presentation. The class will provide the participants with general health information related to exercise, nutrition, and medication management. It includes information on managing flare-ups, working with health care professionals, evaluating treatments, and making informed decisions. This class is already in use in our NIH-funded research [5, 24]. This class serves as a control to the ER class, with matching factors such as duration, structure, format, and location.

Upon completion of study procedures, participants in the control group will be given the option to receive the ER class, and participants in the ER group will continue to have access to the app, but the team will discontinue collecting data on frequency of use.

### Class sites

All treatment sessions will occur at approved clinical or research sites within the Stanford University School of Medicine and Stanford Health Care.

### Instructors

For the ER treatment group, all instructors will be doctoral-level clinical psychologists trained in the treatment of chronic pain. The HE class will be expert-led by experienced health educators or chronic pain professionals (e.g., chronic pain physician assistants).

### Training and monitoring of instructors

ER instructors will be trained in the study protocol for their classes prior to administering treatment. Existing treatment manuals as well as highly structured and standardized class content will assure treatment fidelity. A research coordinator, serving as fidelity rater, will directly observe the first three classes of each treatment arm as well. Cohort effects are likely to be minimal, due to the single-session format and relatively minimal participant interaction.

### Measures

Demographic data, chronic pain history, current and past opioid use, non-opioid medication, and current treatments will be collected at enrollment. The 17-item Current Opioid Misuse Measure [25] will be used to measure opioid misuse and change throughout the study. During screening, we will characterize the patient’s opioid-misuse behaviors using *DSM-5 Opioid* (DSO) [26], a Clinical Trials Network NIDA-supported instrument. Patient-Reported Outcomes Measurement Information System (PROMIS) measures will be used to assess Pain Interference, Physical Function, Depression, Anxiety, Anger, Sleep Disturbance, Fatigue, Social Isolation, and Global Health using short forms [27]. Our group has applied the NIH PROMIS measures in multiple, nationally funded, clinical pain treatment trials and other studies [28–30]. Pain catastrophizing will be assessed using the Pain Catastrophizing Scale (subscales: rumination, magnification, and feelings of helplessness) [31]. Motivational factors, including desire, confidence, readiness, and motivation to reduce opioid use, will be assessed using the questions developed by Goesling and colleagues [32]. Lastly, treatment expectancies will be assessed using the Stanford Expectations of Treatment Scale (SETS) [33].

Baseline period: during screening and enrollment, patients will complete an online baseline assessment with demographics, as well as measures inquiring about pain condition(s) and characteristics, opioid use and misuse, pain catastrophizing, and the PROMIS measures. Within 1 month of starting treatment, participants will also complete 2 weeks of daily surveys assessing daily levels of pain catastrophizing and daily opioid use.

Pre-treatment assessment: Three days pre-treatment, patients will complete an online pre-class survey assessing pain condition and characteristics, opioid use and misuse, pain catastrophizing, and the PROMIS measures. Participants will not be asked to repeat demographic information again, but these measures will be identical to those assessed at baseline.

Post-treatment assessment: immediately post treatment, patients will complete a brief questionnaire assessing patient satisfaction with the intervention on an 11-point Likert scale.

One month post treatment, participants will complete daily surveys assessing daily levels of pain catastrophizing and daily opioid use. For patients in the ER class, they will also complete measures inquiring about daily app and skill use for 2 weeks. At 3, 6, and 12 months post treatment, all participants will complete a set of questionnaires identical to those administered pre-class. The primary study endpoint is 3 months post treatment. App use will also be tracked by REDCap throughout the duration of the trial.

Participants may receive up to US$160 for study completion.

#### Primary outcome measure

Our primary outcome measure is opioid use (domain), which will be converted to morphine-equivalent daily dose (MEDD) (measurement), and assessed as MEDD at baseline, 3-, 6-, and 12-month follow-ups (specific metric). We will report means and standard deviations (method of aggregation) at each time-point and the difference in MEDD within subjects from baseline to the 3-month follow-up time-points (primary follow-up time-point). The difference will be calculated as a percentage difference, and a clinically minimal reduction is defined as > 15% reduction in opioid use in MEDD [34]. We will compare the rate of participants who reach clinically minimal reduction between the two groups. We will quantify absolute opioid reduction in addition to percentage change reduction within subjects and between treatment arms. Finally, we will quantify percentage achieving each group threshold for clinical importance of change (15%, 30%, and 50% as minimally, moderately and substantially important change scores, respectively) [34].

#### Secondary outcome measures

Pain-specific distress (domain) will be assessed using the Pain Catastrophizing Scale [31] (measurement) at baseline, 3-, 6-, and 12-month follow-ups (time-points). We will report the means, range, and standard deviation (specific metric) of the continuous variable (method of aggregation) and compare the change in mean scores (within-subject difference) from baseline to the 3-month follow-up time-point (primary time-point). The mean difference in the ER group will also be compared against the HE arm using the two-sample *t* test. We will calculate the clinical significance of the treatment response as > 30% reduction in pain catastrophizing.

Pain Interference (domain) will be assessed using the PROMIS Pain Interference – short form [35] (measurement) at baseline, 3-, 6-, and 12-month follow-ups (time-points). We will report the mean T-scores and standard deviations of the continuous variable (method of aggregation) and compare the change in mean T-scores (within-subject difference) from baseline to the 3-month follow-up time-point (primary endpoint). The mean difference in the ER group will also be compared against the HE arm using the two-sample *t* test. We will calculate the clinical significance of the treatment response as > 30% reduction in pain catastrophizing.

Opioid misuse (domain) will be assessed using the COMM (Current Opioid Misuse Measure) (measurement) [36] at baseline, 3-, 6-, and 12-month follow-ups (time-points). We will report the means, range, and standard deviation of the continuous variable (specific metric) at baseline, 3-, 6-, and 12-month follow-ups (method of aggregation), and compare the change in mean scores (within-subject difference) from baseline to the 3-month follow-up time-point for primary outcome analysis (time-point). The mean difference in the ER group will also be compared against the HE arm using the two-sample *t* test. We will also compare the proportion of success rate, defined as a reduction to 13 as measured by the COMM. This number is the validated cut-point on COMM [36].

#### Tertiary outcome measures

NIH PROMIS measures [35] (measurement) will be administered to assess Anxiety, Depression, Anger, Fatigue, Physical Function, Global Health, and Sleep Disturbance (domains). These measures have been successfully applied to pain research [13, 37–39]. These measures will be assessed at baseline, 3-, 6-, and 12-month follow-ups (time-points). We will report the mean T-scores and standard deviations of the continuous variable (method of aggregation) and compare the change in mean T-scores (within-subject difference) from baseline to the 3-month follow-up time-point (primary endpoint). The mean difference in the ER group will also be compared against the HE arm using the two-sample *t* test. We will calculate the clinical significance of the treatment response as > 30% reduction in pain catastrophizing.

#### Daily measures

All participants will complete daily measures of opioid medication use, pain intensity, and three-item daily pain catastrophizing scale (PCS) during two 2-week time periods: at baseline (up to 1 month prior to the class), and at follow-up (1month post class). Daily skills’ use will also be assessed for 2 weeks during the follow-up time-period only for the ER treatment arm. These include a four-item questionnaire measuring frequency of use of cognitive, behavioral, or psychophysiological techniques over the past 24 h from 0 times to 5+ times.

### Data collection, quality control, and confidentiality

The online assessments completed by participants will be gathered securely in a REDCap database. Though we do not expect any questionnaires to be collected on paper, if unforeseen circumstances require this to occur, they will be stored as source data and a member of the study team will manually enter the responses into the REDCap database. Additionally, members of the team will be trained to use and complete Case Report Forms (CRFs), how to review them for completeness, as well as how to maintain participant confidentiality. Patient flow will be reported according to the Consolidated Standards of Reporting Trials (CONSORT) guidelines [40].

### Protection of human participants and assessment of safety

#### Protection of human participants

The Stanford University IRB approved this study.

#### Safety monitoring

This trial will be monitored for safety by an independent Data and Safety Monitoring Board (DSMB) composed of a biostatistician and a clinical psychologist with knowledge in treatment of chronic pain conditions. A chairperson of the board will also be appointed who is an individual with expertise in treatment outcome research methodology and who has worked as a consultant on other clinical trial studies and DSMBs. These members will have no other involvement in the study and will serve as independent reviewers of the DSMB. They will convene twice a year or as per needed basis. Members of the DSMB will make relevant safety decisions regarding reported participant cases. The DSMB report will be sent to NIH within 2 weeks of the meeting, twice a year. The report will be sent to the Stanford IRB after meetings have been held for the year, and prior to the continuing renewal.

Members of the DSMB will meet twice per year to review the study’s progress, enrollment, de-identified group-level data for differential rates in key outcomes, and AEs. As a component of the PI’s annual progress report to NIH, she will provide a summary of the DSMB’s reviews and reports. This summary will include sociodemographic data, expected versus actual recruitment rates, treatment retention rates, a description of quality assurance or regulatory issues that occurred during the previous year, a summary of AEs and serious adverse events (SAEs), and any actions or changes regarding the study protocol. The DSM reports to NIH will also include, when available, results of completed data analyses. The DSM reports also will be submitted to Stanford’s IRB prior to beginning the project and, subsequently, at each IRB annual continuing review. Together, the members of the DSMB will review the reports sent by the applicant and will determine whether there is any corrective action, a trigger of an ad hoc review, or stopping-rule violation that should be communicated to Drs. Mackey and Darnall, the PI, the Stanford IRB, and NIH.

In addition, the Advisory Board on the training grant, which is scheduled to meet twice a year will also provide input on these rules. The Advisory Board consists of five experts in study implementation, clinical trial design, opioid measurement and quantification, substance abuse, statistical analyses, and opioid management.

##### Stopping rules

The treatments in this study are not associated with risks. This study will be stopped prior to its completion if: (1) the intervention is associated with adverse effects that call into question the safety of the intervention; (2) difficulty in study recruitment or retention will significantly impact the ability to evaluate the study endpoints; (3) any new information becomes available during the trial that necessitates stopping the trial; or (4) other situations occur that might warrant stopping the trial.

Other issues relating to stopping rules for this study include the development of a detailed protocol for continuous monitoring of AEs in addition to assessing suicide risk and worsening mood, throughout the study period, per the DSMB’s request.

Throughout the duration of the study, each participant will have their PROMIS Depression score calculated at five time-points (at enrollment, 3 days before the class, 3-month follow-up, 6-month follow up, and 12-month follow-up), and if the PROMIS Depression score falls in the severe range (≥ 33), we will employ our safety protocols to ensure patient safety and access to appropriate care.

##### Measurement and reporting of adverse events

Overall, the treatments in this study are not associated with risks. However, we will administer an “Adverse Events Survey”, previously used in one of our clinical trials [5], which assesses any major changes since the last correspondence and covers the following domains: new lifestyle changes, changes in treatment, any positive or negative life events that have impacted their mood or health, any injuries, or illnesses since their last survey. Participants are given the space to provide detail on any of the changes or events that have occurred. This survey is deployed to participants at four time-points, (3 days before the class, and at 3-, 6-, and 12-month follow-up time-points), and these will be assessed weekly by the research coordinator to determine level of risk and take appropriate action. In addition, participants will be encouraged to proactively report any AEs to study staff. All AEs will be recorded on an “Adverse Event Case Report Form.” Adverse events will be discussed in monthly team meetings and will be reviewed and reported to the DSMB. Adverse events will be reported in aggregate to the IRB annually. Known, minor adverse effects will be assessed for, and tracked by, the study coordinator.

Serious adverse events (SAEs): defined according to the Food and Drug Administration (FDA) as any adverse experience that results in any of the following outcomes:

- Life-threatening
- Death
- Hospitalization/prolongation of hospitalization
- Congenital anomaly
- Persistent or significant disability/incapacity
- Required intervention to prevent permanent impairment/damage

The PI will report any SAEs to the Stanford IRB, and NIH. All SAEs will be evaluated by the DSMB and PI within 24 h after the study team becomes aware of the incident. All study-related SAEs will be reported to the NIH within 2 weeks; all others will be included in the annual report to the NIH.

##### Classification of AE severity

Adverse events will be labeled according to severity, which is based on their impact on the patient. An AE will be termed “mild” if it does not have a major impact on the patient, “moderate” if it causes the patient some minor inconvenience, and “severe” if it causes a substantial disruption to the patient’s wellbeing. AEs termed “life-threatening” will be categorized under SAE.

##### AE Attribution Scale

Adverse events will be categorized according to the likelihood that they are related to the study intervention. Specifically, they will be labeled definitely unrelated, definitely related, probably related, or possibly related to the study intervention (see Additional file 1).

### Statistical issues

#### Sample size and detectable differences

We chose our sample size to ensure adequate power to detect treatment effects on the primary outcome (i.e., opioid use) and to investigate pain-catastrophizing reduction as a mediator between the two groups (ER and HE). The project will enroll 136 participants (aged 18–80 years) with diagnosis of chronic non-cancer pain (> 3 months in duration) and currently using prescription opioids.

To compare the main effect of a single-session skills-based pain management class on opioid use against the HE control condition, we will plan to enroll 136 participants and have 116 completers (58 per group). The proposed sample size accounts for 15% attrition in each treatment arm. This is lower than the current attrition rate seen in pain-CBT literature of 18–25% [41, 42], but we believe that our less-burdensome single-session groups will lead to lower rates. We hope to achieve 80% power to detect medium-large treatment effects on the primary outcome (i.e., opioid use).

To examine reduction of PC as a treatment mediator, we will use bias-corrected bootstrapping. Previous data show a large effect of PC treatment on PCS scores (*d* = 0.85–1.15) [4], and others show a medium-size effect of PCS scores on opioid use (*d* = 0.4) [32]. When using bias-corrected bootstrap for mediation analysis, a total of 115 subjects are required for a medium and large effect associations with a mediator variable (PCS score), 80% power, and *α* = 0.05 [43].

### Statistical analyses

#### Primary analyses

We will use an intent-to-treat (ITT) approach in all analyses (i.e., the assessment of individuals will be analyzed by randomized group, regardless of participation in any classes). By doing so, we protect against any confounding that arises as a result of subject dropout.

The main effect of the single-session skills-based pain management class on opioid use will be compared against the HE control class using a two-sample *t* test. Clinically minimal reduction is defined as > 15% reduction in opioid use [34] in MEDD, the recommended unit of measurement in studies of opioid use [44]. We will compare the rate of participants who reach clinically minimal reduction between the two groups. We will quantify absolute opioid reduction in addition to percentage change reduction within subjects and between classes. Finally, we will quantify the percentage achieving each group threshold for importance of change (15%, 30%, and 50% as minimally, moderately, and substantially important change scores, respectively).

To test the hypothesis that the ER class will have greater reductions in pain-related distress, pain interference, and opioid misuse compared to the HE class, our endpoint is opioid misuse, pain catastrophizing, and pain interference at 3 months, and its within-subject difference from baseline is calculated. The mean difference in the ER group will be compared against the HE arm using the two-sample *t* test. We will also compare the proportion of success rate, defined as ≥ 30% reduction in pain catastrophizing and pain interference for clinical significant treatment response [45] and a reduction to 13 as measured by the COMM (Current Opioid Misuse Measure). This number is the validated cut-point on COMM [36].

ITT is also considered conservative in the context of superiority hypothesis testing. A main analysis will be performed of all valid observed data under a plausible assumption about the missing data. This will be followed by sensitivity analyses that accounts for all randomized patients, to explore the effect of departures from the assumption made in the main analysis.

#### Secondary objectives

To test the hypothesis that daily PC predicts same-day and next-day opioid use, a mixed-effects model will be used to study this association. The subject-specific random effects will be used to account for subject-level (level 2) effects; in particular, the effect of the intervention as well as daily-level variations in PC.

Mixed-effects regression will be used to study the association between the 1-month change in daily PC mean and the post-treatment opioid use, the outcome of interest. The regression will adjust for baseline opioid use and other confounding factors. The same analyses will be conducted with opioid misuse, pain intensity, and pain interference as outcomes.

## Discussion

In this trial, we will seek to determine whether a single-session skills-based behavioral pain management class is an effective treatment option for persons with chronic pain who are taking prescription opioids. The study should identify a proportion of patients who achieve a meaningful reduction in opioid use in response to this brief intervention. This will facilitate the future application of a refined version of the class across a variety of settings, such as in primary care or in pre-surgical populations. The study will also elucidate the mechanisms that change opioid use with and without targeted treatment. This information will not only reveal important mechanisms at play but will also allow us to better characterize responders and non-responders to treatment, which will facilitate the development of more tailored and targeted interventions in the future.

### Trial status

NCT03950791 was registered on 10 May 2019. Recruitment began in September 2019. Expected date when recruitment will be completed is 15 May 2023. IRB (protocol #48784) was initially approved on 18 December 2018.

## Supplementary information

**Additional file 1.** Measurement and Reporting of Adverse Events

## Data Availability

Data will be available on ClinicalTrials.gov (NCT03950791).
