# Supplementary material for: Efficacy and mechanisms of a single-session behavioral medicine class among patients with chronic pain taking prescription opioids: study protocol for a randomized controlled trial
Source: Trials. 2020 Jun 12;21:521. doi: 10.1186/s13063-020-04415-x (PMC7290153; doi:10.1186/s13063-020-04415-x)
Supplement: Supplementary file 1 — Additional file 1. Measurement and Reporting of Adverse Events [file 13063_2020_4415_MOESM1_ESM.doc]

**FOR QUESTIONS ABOUT THE STUDY, CONTACT:** Dr. Maisa Ziadni, Stanford Systems Neuroscience and Pain Laboratory, 1070 Arastradero, Suite 200, 2C2728, Palo Alto, CA 94304. Phone number is (650) 736-5494.

DESCRIPTION: The purpose of this research is to understand how pain psychology classes and health education information may reduce opioid use. You will be asked to come to one class session, answer daily questions, and complete questionnaires. We ask that you refrain from new behavioral pain treatments during the study period. However, you may continue to seek standard pain treatment.

Class sessions: You may be randomly assigned to one of the two educational groups described below. You cannot be reassigned once you are allocated to a treatment.

i. Pain psychology class is a 2-hour pain psychology education class. You will learn various information and skills to better manage important aspects of your pain. For the pain psychology class participants, there is an optional application you may download with a relaxation resource. We will only collect application data on the frequency of use and length of use.

ii. Health education class is an approximately 2-hour class that will cover information important to your health and pain.

Throughout the study:

i. Daily questionnaires about your pain and mood to be completed twice for a period of 2 weeks before and 2 weeks after your class. This typically takes less than 5 minutes to complete each day and will be completed through a text or email.

ii. We may ask you to fill out questionnaires at different time points during the study: before treatment start and after treatment at 3, 6, and 12 months. These questions may take approximately 20-30 minutes to complete and include questions about your symptoms, mood, function, medication use, and other treatments being used. This may occur via secure web-survey, regular mail, or by phone interview.

**RISKS AND BENEFITS:**

The risks associated with this study are:

- There is a risk you may feel uncomfortable answering some of the questions on the questionnaires. You have the right to refuse to answer particular questions.
- It is possible that, based on information gained from this study, the investigators may be required to report information (e.g., information relating to suicide, physical or sexual abuse) to the appropriate authorities.
- During the class it is possible that you may experience discomfort related to observing your thought and emotions when practicing the skills in the intervention. You may also find it difficult to complete assignments to practice the skills at home. Although this training may benefit most people, some people may experience an unforeseeable negative reaction or minimal reaction to the methods and skills taught in these interventions.

The benefits which may reasonably be expected to result from this study are that you may experience pain relief from the techniques and methods you learn in your classes. We cannot and do not guarantee or promise that you will receive any benefits from this study. Your decision whether or not to participate in this study will not affect your employment/medical care.

**TIME INVOLVEMENT:** Your total participation in this experiment will take approximately 12 months.

**PAYMENTS:** You may receive up to $160 as payment for your participation in the study, which includes completing the daily tracking and questionnaires for the entire 12 months post-class period. If you will not complete the study, you will only be paid for those visits and activities which you have actually attended or performed.

Payments may only be made to U.S. citizens, legal resident aliens, and those who have a work eligible visa. You may need to provide your social security number to receive payment.

**PARTICIPANT’S RIGHTS:** If you have read this form and have decided to participate in this project, please understand your participation is voluntary and you have the right to withdraw your consent or discontinue participation at any time without penalty or loss of benefits to which you are otherwise entitled.

The results of this research study may be presented at scientific or professional meetings or published in scientific journals. However, your identity will not be disclosed**.** You have the right to refuse to answer particular questions.

This research is covered by a Certificate of Confidentiality from the National Institutes of Health. The researchers with this Certificate may not disclose or use information, documents, or biospecimens that may identify you in any federal, state, or local civil, criminal, administrative, legislative, or other action, suit, or proceeding, or be used as evidence, for example, if there is a court subpoena, unless you have consented for this use. Information, documents, or biospecimens protected by this Certificate cannot be disclosed to anyone else who is not connected with the research except, if there is a federal, state, or local law that requires disclosure (such as to report child abuse or communicable diseases but not for federal, state, or local civil, criminal, administrative, legislative, or other proceedings, see below); if you have consented to the disclosure, including for your medical treatment; or if it is used for other scientific research, as allowed by federal regulations protecting research subjects.

The Certificate cannot be used to refuse a request for information from personnel of the United States federal or state government agency sponsoring the project that is needed for auditing or program evaluation by The National Institute on Drug Abuse which is funding this project or for information that must be disclosed in order to meet the requirements of the federal Food and Drug Administration (FDA).  You should understand that a Certificate of Confidentiality does not prevent you from voluntarily releasing information about yourself or your involvement in this research. If you want your research information released to an insurer, medical care provider, or any other person not connected with the research, you must provide consent to allow the researchers to release it.

The Certificate of Confidentiality will not be used to prevent disclosure for any purpose you have consented to in this informed consent document, including information you provide regarding your medication use, drug use, or pain levels.

**Authorization To Use Your Health Information For Research Purposes**

Because information about you and your health is personal and private, it generally cannot be used in this research study without your authorization. If you agree to this form, it will provide that authorization. The form is intended to inform you about how your health information will be used or disclosed in the study. Your information will only be used in accordance with this authorization form and the informed consent form and as required or allowed by law. Please read it carefully before agreeing to it.

**What is the purpose of this research study and how will my health information be utilized in the study?**

The purpose of this research study is to compare the efficacy of 2 different behavioral interventions in improving the symptoms of pain and reducing the need for pain medication. Information we collect from you will be used to evaluate the effectiveness of the class, and may be used in publications as well.

**Do I have to agree to this authorization form?**

You do not have to agree this authorization form. But if you do not, you will not be able to participate in this research study, including receiving any research-related treatment.

Agreeing to the form is not a condition for receiving any medical care outside the study.

**If I agree, can I revoke it or withdraw from the research later?**

If you decide to participate, you are free to withdraw your authorization regarding the use and disclosure of your health information (and to discontinue any other participation in the study) at any time. After any revocation, your health information will no longer be used or disclosed in the study, except to the extent that the law allows us to continue using your information (e.g., necessary to maintain integrity of research). If you wish to revoke your authorization for the research use or disclosure of your health information in this study, you must write to: Maisa Ziadni, PhD, or Sean Mackey PhD, at 1070 Arastradero Road, Suite 200, Palo Alto, CA 94304.

**What Personal Information Will Be Obtained, Used or Disclosed?**

Your health information related to this study, may be used or disclosed in connection with this research study, including, but not limited to, your name, contact information, date of birth, demographics, medical and pain history, psychological history, pain intensity, pain catastrophizing scale scores, and medication use. We may also collect responses to questionnaires and daily questions information, and treatment satisfaction measures.

**Who May Use or Disclose the Information?**

The following parties are authorized to use and/or disclose your health information in connection with this research study:

- The Protocol Directors Sean Mackey, MD, PhD and Maisa Ziadni, PhD.
- The Stanford University Administrative Panel on Human Subjects in Medical Research and any other unit of Stanford University as necessary
- Research Staff

**Who May Receive or Use the Information?**

The parties listed in the preceding paragraph may disclose your health information to the following persons and organizations for their use in connection with this research study:

- The Office for Human Research Protections in the U.S. Department of Health and Human Services
- The National Institute on Drug Abuse (NIDA)

Your information may be re-disclosed by the recipients described above, if they are not required by law to protect the privacy of the information.

**When will my authorization expire?**

Your authorization for the use and/or disclosure of your health information will end on December 31^st^, 2060, or when the research project ends, whichever is earlier.

**WITHDRAWAL FROM STUDY**

The Protocol Director may also withdraw you from the study without your consent for one or more of the following reasons:

- - Failure to follow the instructions of the Protocol Director and study staff.
  - The Protocol Director decides that continuing your participation could be harmful to you.
  - You need treatment not allowed in the study.
  - The study is cancelled.
  - Other administrative reasons.
  - Unanticipated circumstances.

**Contact Information:**

If you have any questions, concerns or complaints about this research study, its procedures, risks and benefits, or alternative courses of treatment, you should ask the Protocol Director, Sean Mackey, MD, PhD at (650) 725-9636 or Maisa Ziadni PhD, at (650) 736-5494. You should also contact them at any time if you feel you have been hurt by being a part of this study.

Independent Contact: If you are not satisfied with how this study is being conducted, or if you have any concerns, complaints, or general questions about the research or your rights as a participant, please contact the Stanford Institutional Review Board (IRB) to speak to someone independent of the research team at (650)-723-5244 or toll free at 1-866-680-2906.  You can also write to the Stanford IRB, Stanford University, 3000 El Camino Real, Five Palo Alto Square, 4th Floor, Palo Alto, CA 94306.

By providing your verbal consent, you are agreeing to participate in the research study.

A copy of this consent form will be emailed to you for your personal records.
